# Innovative education for adolescent mothers in neonatal care: practices in nursing training*

**DOI:** 10.1590/1980-220X-REEUSP-2025-0499en

**Published:** 2026-05-11

**Authors:** Maria Cecília Bandeira Arnaud Moura, Ana Maria Cavalcante Melo, Pedro Henrique do Nascimento Silva, Mércia Lamenha Medeiros

**Affiliations:** 1 Universidade Federal de Alagoas, Faculdade de Medicina, Maceió, AL, Brazil.; 2 Universidade Federal de Pernambuco, Recife, PE, Brazil.

**Keywords:** Maternal and Child Health, Education, nursing, Professional Training, Adolescent Mothers

## Abstract

**Objective:**

To develop the skills of undergraduate nursing students for health education with adolescent mothers.

**Method:**

Qualitative study, action research, public federal university hospital, with undergraduate nursing students in their neonatal internship, and with adolescent mothers. The interventions were established through the creation of a practical guide for safe discharge, awareness workshops for graduating students and educational sessions with mothers, conversation circles, and realistic simulations in an appropriate space, with feedback from the students and mothers. Data were analyzed using informational clusters, thematic analysis, and categorization.

**Results:**

The graduating students recognized the importance of educational initiatives for teenage mothers, and the benefit to their development. Active methodologies can strengthen reflective practice and training in undergraduate nursing programs, promoting playful strategies for self-efficacy in caregiving among adolescent mothers. The intervention promoted the empowerment of teenage mothers through the listening to their needs and fostered their autonomy.

**Conclusion:**

Innovative and interactive education can promote meaningful learning, improve professional qualifications, and align teaching with social demands, with a focus on health education for adolescent mothers.

## INTRODUCTION

Adolescence encompasses the transition between childhood and adulthood, a phase of discovery and the pursuit of autonomy. This phase can lead to increased susceptibility to risky situations, and both objective and subjective aspects of the context in which adolescents live can influence them. This vulnerability exposes them to conditions that compromise their health and to risky behaviors, such as unwanted pregnancies, unprotected sex, infections, and other health problems^(1)^.

Therefore, justifying vulnerability solely based on biological aspects is a highly reductionist approach that fails to consider psychological and socio-environmental factors. Simply associating vulnerability with the idea of youthful rebellion can reinforce the public stereotype of hostility towards teenagers. The model to be followed by healthcare should allow for discussion about preventative behaviors and the development of skills by adolescents^(2)^.

From this perspective, and considering the complexity of the adolescence process, health professionals working through managerial, care, and educational actions, both individually and collectively, in different settings, face constant challenges in developing and applying health education strategies aimed at facilitating the educational process with adolescents^(3)^.

In the context of undergraduate Nursing programs, it is essential that students develop skills to work in health education, especially with vulnerable populations, such as adolescent mothers.

Meaningful learning occurs when new knowledge is integrated into the student’s cognitive structure, becoming relevant and applicable in practice. The importance of dialogue and problematization in the educational process is highlighted, emphasizing that teaching should be an emancipatory act in which educator and learner learn together in a continuous process^(4)^.

In this context, neonatal nursing professionals frequently deal with the uniqueness of their practice, marked by a collaboration peculiar to the field. The practical experience in providing care makes the inclusion of this professional fundamental in training, knowledge sharing, and the development of skills that enable good maternal choices in newborn care^(5)^.

It is known that the prenatal care guidance received by Brazilian women emphasizes information about referral maternity hospitals, but there is inequity in the provision of this guidance^(6)^.

Within the context of hospital care, health education strategies focus on infant care, guidance on breastfeeding, and women’s health in the postpartum period. Applying these strategies during the hospitalization period can help reduce anxiety in adolescent mothers, especially those of premature babies. Additionally, it promotes greater confidence in the continuity of care at home, increases adherence to outpatient follow-up, and reduces the frequency of new hospitalizations and/or deaths^(7)^.

According to this theoretical approach, the present research aimed to develop skills in undergraduate nursing students for health education with adolescent mothers, as a way to empower these mothers to take a leading role in the care of their own children.

## METHOD

### Design of Study

This research uses a qualitative approach, guided by the action research method. The action research design, being a type of empirically based social research, was conceived and carried out in close association with an action or the resolution of a collective problem, in which researchers and participants are involved in a cooperative or participatory manner. The analysis of the object of identification enables the construction of knowledge; it has all the requirements and instruments to be considered a scientific construct^(8)^.

### Population, Local and Selection Criteria

The research was conducted in a Neonatal Unit at the University Hospital maternity ward, a federal public hospital, involving undergraduate Nursing students in supervised curricular internships and adolescent mothers, aged between 14 and 18 years, who accompanied their newborns in the same hospital unit. The sample consisted of undergraduate nursing students enrolled in a supervised curricular internship (*ECS*) in a hospital setting. The selection of this group was justified by the intensive immersion of these students in clinical practice, since the internship comprises a workload of 500 hours, completed in one academic semester, as recommended by the National Curriculum Guidelines (DCN) for the Nursing course^(9)^. Specifically, the participants selected the Neonatal Unit as their field of practice. Allocation to this unit is limited to three places per semester, with the selection process governed by institutional criteria that consider the student’s affinity with the area and, secondarily, academic performance as a tie-breaker. The teenage mothers had already been discharged from obstetrics and were accompanying their hospitalized newborns; the type of delivery did not influence their participation, and all were available during their children’s hospitalization period, which varied according to their clinical condition.

Considering the nature of action research and the study setting, the chosen scenario was the Kangaroo Neonatal Intermediate Care Unit (*UCINCA*), which currently has 5 beds. The sample selection was carried out respecting the saturation process in the narratives. This procedure allows defining the final sample size from the moment when new inclusions cease to add relevant information to the phenomenon being investigated, thus stopping the recruitment of new participants and the holding of new workshops^(10)^. In qualitative research, the sample size must be linked to the scope of the research object (or question), which, in turn, is connected to the choice of the group or groups to be interviewed and monitored through participant observation^(11)^.

### Data Collection

The research was conducted over a period of 3 months. It was subdivided into 3 phases, during the months of August and November in the year 2024. Data collection was performed by this study’s principal investigator.

The educational intervention for undergraduate students and teenage mothers consisted of sharing prior experiences, addressing doubts, clear communication, and adapting language to the local context, using active teaching-learning methodologies (practical workshop, realistic simulation, brainstorm, and interactive presentations with videos), grounded in the principles of andragogy under the light of Ausubel’s theory of meaningful learning^(12)^.

The first stage, lasting 2 hours, was a conversation circle with the undergraduate students, during which the Practical Guide for the Safe Hospital Discharge of Newborns, previously prepared by the researchers, was made available. The students were encouraged to share ideas to help build useful knowledge for teenage mothers, ensuring the safe discharge of their children. At each stage, an evaluation and a feedback regarding the Practical Guide, based on their knowledge and experience, were carried out. The following week, the second stage took place, lasting 3 hours. The Realistic Simulation Workshop with the students used simulations with dolls to demonstrate the information contained in the Practical Guide. Attempting to recreate a welcoming and functional environment, similar to the homes of teenage mothers, this stage led to changes in the guide and recording of the narratives. The 3^rd^ stage took place over 2 days with a 15-day interval between them, lasting 2 hours and 10 minutes and 2 hours and 40 minutes respectively. It consisted of a Realistic Simulation Workshop with teenage mothers and was conducted by the students under the supervision of the principal investigator. The guide was a tool within the active teaching-learning process, with simulated practice followed by the reproduction of the learned care practices by the teenagers, using dolls. The aim was to promote the development of skills and knowledge building among adolescent mothers. This stage generated the final version of the guide *VAMOS PRA CASA, MAMÃE?* (Let’s go home, Mom?), validated during the approval phase of the main author’s Master’s thesis.

### Data Analysis and Treatment

A recorder was used to capture the participants’ narratives, and then the speeches were transcribed. They were evaluated using thematic content analysis, following these steps: pre-analysis, exploration of the material, processing of the results obtained, and interpretation^(13)^. After transcribing the recorded narratives and exhaustive readings by the researchers, a summary table was created for the thematic classification and categorization.

### Ethical Aspects

The study followed all ethical precepts after being approved by the Ethics Committee, CAAE 71297523.10000.5013 and approved opinion no. 6,388,587, on October 4, 2023. To ensure anonymity for participants, they were identified by the letters “E” for interns (*E*stagiário in Portuguese) and “A” for adolescent mothers, followed by a cardinal number (e.g., E1, E2, A1, A2) when referring to group discussions.

## RESULTS AND DISCUSSION

The analysis included 3 undergraduate nursing students and 4 adolescent mothers (14–18 years old) during their newborns’ stay in the hospital unit.

The narratives analysis inferred four thematic categories, namely: 1 – Reflective Practice and the Teaching-Learning Process; 2 – Strategies for the educational process of adolescent mothers: playful and humanized approaches; 3 – Improvement of Nursing Practice; 4 – Maternal Empowerment: Feedback as a Learning Tool in Nursing.

### Category 1: Reflective Practice and the Teaching-Learning Process

Acquiring skills and competencies in nursing requires that workplace learning be based on reflection and understanding of the context of the adolescent mothers being cared for. For the graduate students, direct interaction with these mothers highlighted the need to adapt the content to individual specificities, promoting sensitive and adaptable teaching.

During the educational intervention, challenges such as low educational attainment and socioeconomic conditions demanded alternative strategies, such as visual aids and clinical simulations. These aspects reinforce the importance of reflective practice, which includes understanding the social determinants of health^(3)^.

Training based on critical reflection and practical experience is essential for developing empathy and caregiving skills, reducing maternal stress and strengthening parental autonomy^(14)^. The educational intervention implemented provided a deeper understanding of the challenges faced by adolescent mothers, allowing graduate students to enhance their ability to actively listen and adapt to each family’s context.

*I think it’s the ability to adapt to the mother and the context she lives in, what she has at home, in fact… what we teach isn’t always what she has available at home. (E3)*

Clinical simulations were fundamental to the development of empathy and social commitment among Nursing students, aligning with pedagogical guidelines that promote collaborative and inclusive learning^(15)^. Empathy, defined as the psychological capacity to understand the emotions and realities of others, is an essential skill that should be developed through reflective training^(16)^.

*This training helped me think about how I’m going to talk to people, depending on their social level and cognitive comprehension as well (E1). We need to find another way to understand it, especially because the audience has diverse levels of understanding. (E1)*

In the educational context, studies indicate that integrating practical activities into curricula is effective in mitigating prejudice and improving interaction with vulnerable populations^(17)^. It is also worth highlighting that learning in real-world settings promotes person-centered care, an essential aspect for the training of nurses^(18)^.

*Language and non-judgment are very important, because she thinks she is not capable because she had a child young[…] we have to be ready to help […] (E2.) […]If I hadn’t taken the workshop, I wouldn’t know how to apply it and teach other mothers (E1). Allow her to be an active subject, participating in this care[…] (E3).*

This study reinforced the idea that teaching and learning in Nursing should be centered on the individual (in this case, the undergraduate student and adolescent mother), promoting collaboration and the development of skills aimed at humanizing care. Student motivation is an essential factor for the effectiveness of this process.

### Category 2: Strategies for the Educational Process of Teenage Mothers: Playful and Humanized Approaches

Developing empathic skills and adopting educational strategies adapted to the life context of adolescent mothers are essential for reflective practice and an effective teaching-learning process. This was evidenced in this study.

*[…] adolescent, he is constantly changing and depending on the stimulus he receives, which will determine how he will act or not (E2).*

The experience of Nursing graduates revealed that a welcoming and judgment-free environment encourages mothers to share their doubts and difficulties.

*They keep thinking they’ll be judged if they don’t do the right thing (E3).*

The diversity of experiences among the participants reinforces the need to adapt educational strategies, as exemplified by the creation of visual materials to facilitate understanding for mothers with low levels of education. Studies indicate that the use of playful and interactive methodologies, such as practical simulations with dolls and other teaching materials, promotes meaningful learning and confidence among adolescent mothers in caring for their children^(19)^.

Furthermore, studies also reinforce the importance of developing specific educational materials for this population, promoting autonomy and confidence in decision-making^(20)^.

*[…] We could draw a picture of a thermometer… Put it down there that anything in green is okay, anything in red or different is a warning. Because then she would look at the thermometer and even if you don’t know how to read it, you’ll compare, see if it’s the same (E2).*

The transition to motherhood in adolescence is permeated by intersectional vulnerabilities that can impact the maternal experience, influenced by the social space occupied by the mother and the father^(21)^. The support network, while essential, can also hinder the autonomy of adolescent mothers, as observed in the account of one of the nursing graduates.

*I think the teenage girl loses her role as a mother, and the grandmother takes over that role. Her mother is also the child’s mother, which could not happen (E1).*

The implementation of Educational Technologies (ET) emerges as an effective alternative to expand the possibilities of health education, offering materials and strategies that enhance learning and encourage the active participation of adolescent girls^(20)^. Traditional methodologies tend to reinforce the learners’ passivity, making it necessary to use innovative approaches that promote more dynamic and engaging interactions^(19)^.

*Practice is best for her to learn by doing. To feel part of the process. (E2) They are attentive to details (E3).*

*And the more you apply it, the more you learn. Each new workshop offers a different learning experience. One mother went one way and the other went another way (E1).*

Nursing professionals must be prepared to meet the specific needs of pregnant adolescents, providing support, guidance, and empathy^(22)^.

The research reinforces the importance of humanized and interactive educational practices that value the experiences of adolescent mothers and strengthen their leading role in caring for their children, promoting greater confidence and autonomy during motherhood.

### Category 3: Improvement of Nursing Practice

Enhancing the practice for undergraduate Nursing students involved in educational interventions with adolescent mothers is a process of mutual construction, in which future nursing professionals and adolescent mothers develop their learning together.

The participation of undergraduate students in an educational intervention on safe discharge of newborns, combined with the application of a practical guide, allowed the integration of prior knowledge with new concepts, promoting meaningful learning^(12)^.

The theoretical learning of the undergraduates in the intervention was complemented by realistic simulations, fundamental for evidence-based practice, which reinforce the competencies and skills of the undergraduates, preparing them for patient care.

*[…] We saw here what needs to be done, we saw in the manual how to do it, we saw how to do it in practice, we put it into practice, we watched what we read before, we already connected it with that and now I’m redoing it. This adds more knowledge[…] (E3).*

Evidence-based Nursing practice integrates the best available tools, the educator’s experience, and the patients’ needs^(23)^.

Realistic simulation helps bridge the gap between theoretical learning and practice, thus improving understanding. Interacting with teenage mothers was a process in which graduate students adapted their educational approaches to the specific needs of the mothers, considering their socioeconomic and emotional context, which reinforced their communication and empathy skills^(24)^.

*So, understanding how teenagers learn, a little more about neuroscience. It makes us open our eyes to understand what happens in their brains, to embrace them and try to reach them in a way (E1).*

Therefore, Nursing education involves a dynamic learning process aimed at technical competence and adaptation to different scenarios.

### Maternal Empowerment: Feedback as a Learning Tool in Nursing

The educational intervention with adolescent mothers, conducted through group discussions and realistic simulations, fostered appreciation and meaningful learning about newborn care. The supportive approach, combined with the use of dolls to simulate safe discharge practices, facilitates the acquisition of skills by mothers, promoting confidence and autonomy from the beginning of motherhood^(25)^.

*[…]because, whether we like it or not, the feeling of belonging will empower them so that when someone at home says: No, girl! There’s no need for that” …they are able to show that they know (E3).*

This intervention provided a supportive and learning space that eliminated insecurity and increased teenage mothers’ self-confidence^(26)^.

*It was important…And also to show people outside that I am capable of what they think I won’t be capable of (A3). It would also be good if it were at discharge (A2). It says a lot of things I didn’t know. Whether single or married, that I didn’t have much support. Then, go ahead and forget about something, that’s good, right? Or even a first-time mother. Yes, first-time mom, that’s when you get lost (A3).*

The health education process is based on raising individual awareness, which empowers mothers to act safely and autonomously^(27)^. In an integrative review, the authors reflected on the importance of health education for adolescent mothers, highlighting the need to begin this education during prenatal care, in an individualized manner, considering the mothers’ social, cultural, emotional, and educational context. This facilitates the understanding of information and reduces parental stress^(28)^.

The use of feedback from the adolescent mothers, along with faculty supervision, was essential to adjusting the teaching-learning process, personalizing care, and strengthening competencies in Nursing students. Morin emphasizes that health education should be reflective and transformative, which was facilitated by integrating technical knowledge with the reality of adolescent mothers^(29)^.

*It would be good if they did it regularly, because it’s important to keep remembering, right? The important things (A3).*

*But I liked reading first and then doing (A2).*

The continuous feedback was fundamental to enhancing teaching practice and understanding of care^(30)^.

*I think it’s a combination. We are better prepared and with that they have a better level of understanding (E2).*

*Both professionally, educationally, and interpersonally. You learn to look at the other person. You see the other person in a vulnerability that you wouldn’t have imagined (E1)*.

The study results showed that ongoing dialogue between teenage mothers and graduate students was crucial for obtaining feedback and adjusting teaching strategies. The learner’s leading role was emphasized, contributing to better performance by the graduating students and promoting a greater understanding of teenage mothers. These exchanges were essential for adapting educational approaches, resulting in a more effective and satisfactory teaching-learning process for both parties involved.

### Limitations and Perspectives

This study has limitations related to the fact that it was developed in a single hospital setting, which restricts the transferability of the findings to other contexts. Added to this are possible biases inherent in qualitative research, especially the bias of subjectivity, considering that the interventions were conducted by undergraduate Nursing students, whose perceptions and practices are influenced by prior experiences, expectations, and levels of academic maturity. The direct involvement of undergraduate students in the educational process and the professional ties of some of the researchers to the sector may have contributed to a positive valuation of the experiences lived, characterizing a possible bias towards involvement.

The sex issue (only female nursing students were present in the study) stems from the prevalence of women in the Nursing course. To minimize biases, strategies were adopted such as having only one researcher from the sector present during the workshops, listening to the undergraduate students beforehand before the interventions, using a pedagogical physical space outside the Neonatal Unit, and having the newborns supervised by the Nursing team during the activities. The short duration of the supervised internship may also have impacted the continuity of interventions and the longitudinal monitoring of the adolescent mothers’ learning. Looking to the future, it is recommended to extend the follow- up period, use data triangulation strategies, and expand educational activities to other levels of healthcare, especially Primary Care.

In summary, the results presented do not exhaust the investigated problem, constituting a provisional synthesis of the phenomenon. The evidence gathered in the field, in dialogue with the literature, raises new questions that transcend the scope of this study. Under the paradigm of qualitative research, it is understood that scientific knowledge is built through successive approximations, establishing a basis for future investigations to deepen the discussions outlined here.

## CONCLUSION

The mothers’ engagement in the educational process improved their confidence in caring for their newborns and reinforced the importance of health education as a tool for empowering adolescent girls and promoting social transformation.

The findings of this study highlight the relevance of integrating innovative methodologies and educational technologies into the Nursing curriculum, ensuring training that is more aligned with social demands. Realistic simulation and the use of visual materials have proven to be valuable tools in facilitating learning, allowing future professionals to have a more solid preparation for maternal and child care. Thus, the undergraduates’ experience demonstrated the positive impact of interactive education on the qualification of professionals and the improvement of care provided.

The need for continued and expanded research on educational practices aimed at vulnerable populations was reinforced, especially among adolescent mothers. Adapting teaching strategies to different sociocultural contexts contributes to the training of nurses and also to the promotion of more inclusive and effective care. The association between theory, practice, and critical reflection can be crucial for improvements in nursing education, ensuring professionals are trained to act with sensitivity, ethics, and commitment to maternal and child health.

## Data Availability

As recommended by Open Science, the link to the repository where the research data was deposited is: https://famed.ufal.br/pt-br/pos-graduacao/ensino-na-saude/documentos/taac/2025/tacc_para_sigaa_23-05.pdf/view.
