# Supplementary material for: Innovative education for adolescent mothers in neonatal care: practices in nursing training
Source: Rev Esc Enferm USP. 2026 May 11;60:e20250499. doi: 10.1590/1980-220X-REEUSP-2025-0499en (PMC13160411; doi:10.1590/1980-220X-REEUSP-2025-0499en)
Supplement: Translation

# Translation: Educação inovadora para mães adolescentes nos cuidados neonatais: práticas na formação da enfermagem*

**DOI:** 10.1590/1980-220X-REEUSP-2025-0499pt

**Authors:** Maria Cecília Bandeira Arnaud Moura, Ana Maria Cavalcante Melo, Pedro Henrique do Nascimento Silva, Mércia Lamenha Medeiros

**Affiliations:** 1 Universidade Federal de Alagoas, Faculdade de Medicina, Maceió, AL, Brasil.; 2 Universidade Federal de Pernambuco, Recife, PE, Brasil.

**Keywords:** Saúde Materno-Infantil;Educação em Enfermagem, Capacitação Profissional, Mães Adolescentes

## Abstract

**Objetivo:**

Desenvolver habilidades de graduandos de Enfermagem para educação em saúde com mães adolescentes.

**Método:**

Estudo qualitativo, pesquisa-ação, hospital universitário, público, federal, com graduandas de Enfermagem, no estágio Neonatal, e com mães adolescentes. As intervenções estabeleceram-se pela construção do guia prático para alta segura, oficinas de sensibilização das graduandas e educativa com as mães, roda de conversa e simulação realística, em espaço apropriado, com feedbacks das graduandas e das mães. Os dados foram analisados em núcleos informativos, análise de temática e categorizados.

**Resultados:**

As graduandas reconheceram a importância de ações educativas para as mães adolescentes, e o benefício em sua formação. Metodologias ativas podem fortalecer a prática reflexiva e a formação na graduação de Enfermagem, promover estratégias lúdicas para a autoeficácia no cuidado, em mães adolescentes. A intervenção promoveu a valorização das mães adolescentes por meio da escuta das demandas e favoreceu a autonomia.

**Conclusão:**

A educação inovadora e interativa pode promover aprendizagem significativa, melhorar a qualificação profissional e alinhar o ensino às demandas sociais, com atenção à educação em saúde de mães adolescentes.

## INTRODUÇÃO

A adolescência compreende transição entre a infância e a vida adulta, fase de descobertas e busca por autonomia. Tal fase pode trazer maior suscetibilidade a situações de risco, influenciando tanto por aspectos objetivos quanto subjetivos no contexto em que vivem os adolescentes (Podem vir a ser mais suscetíveis a situações de risco, influenciados tanto por aspectos objetivos quanto subjetivos no contexto em que vivem). Essa vulnerabilidade os expõe a condições que comprometem a saúde e a comportamentos de risco, como gestações indesejadas, sexo desprotegido, infecções e outros agravos^(1)^.

Portanto, justificar a vulnerabilidade apenas aos aspectos biológicos é muito reducionista se não levar em consideração aspectos psicológicos e socioambientais. A associação da vulnerabilidade, simplesmente, à ideia de rebeldia jovem, pode reforçar o estereotipo público de hostilidade aos adolescentes. O modelo a ser seguido pela saúde deve permitir a discussão sobre comportamentos preventivos e o desenvolvimento de habilidades pelos adolescentes^(2)^.

Sob essa perspectiva e considerando a complexidade do processo de adolescer, os profissionais de saúde que atuam por meio de ações gerenciais, assistenciais, educacionais, de âmbito individual e coletivo, em diferentes espaços, enfrentam constantes desafios para desenvolver e aplicar estratégias de Educação em Saúde, que visem facilitar o processo educacional com adolescentes^(3)^.

No contexto da graduação em Enfermagem é essencial que os estudantes desenvolvam competências para atuar na educação em saúde, especialmente com populações em situação de vulnerabilidade, como mães adolescentes.

Aprendizagem significativa ocorre quando novos conhecimentos são integrados à estrutura cognitiva do estudante, tornando-se relevantes e aplicáveis na prática. Destaca-se a importância do diálogo e da problematização no processo educativo, enfatizando que o ensino deve ser um ato emancipador, no qual educador e educando aprendem juntos em um processo contínuo^(4)^.

Nesse contexto, os profissionais de enfermagem em Neonatologia frequentemente lidam com a singularidade de sua prática, marcada por uma colaboração peculiar à área. A experiência concreta no manejo assistencial torna a inserção desse profissional fundamental na formação, o compartilhamento de conhecimentos e o desenvolvimento de habilidades que possibilitem boas escolhas maternas nos cuidados aos recém-nascidos^(5)^.

Sabe-se que as orientações recebidas no pré-natal pelas mulheres brasileiras se destacam para orientações sobre maternidade de referência, mas há iniquidade na oferta de orientações^(6)^.

No âmbito da assistência hospitalar, as estratégias de educação em saúde direcionam-se para o cuidado com o bebê, a orientação sobre aleitamento materno e a saúde da mulher no puerpério. A aplicação dessas estratégias durante o período de hospitalização pode contribuir para reduzir a ansiedade de mães adolescentes, em especial de prematuros. Adicionalmente, promove maior confiança para a continuidade dos cuidados no domicílio, aumenta a adesão ao acompanhamento ambulatorial e reduz a frequência de novas internações e/ou óbitos^(7)^.

Segundo essa abordagem teórica, a presente pesquisa, visou desenvolver habilidades em graduandos de Enfermagem para educação em saúde com mães adolescentes, como forma de tornar essas mães protagonistas no cuidado do próprio filho.

## MÉTODO

### Desenho do Estudo

Pesquisa de abordagem qualitativa, norteada pelo método pesquisa – ação. O desenho pesquisa – ação, por ser um tipo de pesquisa social com base empírica, foi concebido e realizado em estreita associação com uma ação ou com a resolução de um problema coletivo e no qual pesquisadores e participantes estão envolvidos de modo cooperativo ou participativo. A análise do objeto de identificação possibilita a construção do conhecimento, possui todos os requisitos e instrumento para ser considerado um construto científico^(8)^.

### Local, População e Critérios de Seleção

A pesquisa foi realizada em uma Unidade Neonatal na maternidade Hospital Universitário, público federal, envolvendo graduandas de enfermagem, em estágio curricular supervisionado e mães adolescentes, com idades entre 14 e 18 anos, que acompanharam seus recém-nascidos na mesma unidade hospitalar. A amostra foi composta por discentes de graduação em Enfermagem inseridas no Estágio Curricular Supervisionado (ECS) em ambiente hospitalar. A escolha desse grupo justificou-se pela imersão intensiva dessas discentes na prática assistencial, visto que o estágio compreende uma carga horária de 500 horas, integralizadas em um semestre letivo, conforme preconizado pelas Diretrizes Curriculares Nacionais (DCN) para o curso de Enfermagem^(9)^. Especificamente, as participantes selecionaram a Unidade Neonatal como campo de prática. A alocação nesta unidade é limitada a três vagas por semestre, sendo o processo de seleção regido por critérios institucionais que consideram a afinidade da discente com a área e, subsidiariamente, o desempenho acadêmico como critério de desempate. As mães adolescentes já haviam recebido alta obstétrica e acompanhavam seus recém-nascidos internados; o tipo de parto não influenciou a participação, e todas estavam disponíveis durante o período de internação dos filhos, que variou conforme a condição clínica.

Considerando a natureza da pesquisa-ação e o cenário do estudo, o cenário escolhido foi a Unidade de Cuidados Intermediários Neonatal Canguru (UCINCA), que atualmente comporta 5 leitos. A seleção da amostra ocorreu respeitando o processo de saturação nas narrativas, esse procedimento permite definir o tamanho final da amostra a partir do momento em que novas inclusões deixam de acrescentar informações relevantes ao fenômeno investigado, interrompendo-se a captação de novos participantes e realização de novas oficinas^(10)^. A amostra em uma pesquisa qualitativa deve estar vinculada à dimensão do objeto (ou do questionamento) que, por sua vez, se articula com a escolha do grupo ou dos grupos a serem entrevistados e acompanhados por observação participante^(11)^.

### Coleta de Dados

O desenvolvimento da pesquisa ocorreu ao longo de 03 meses, subdividida em 03 momentos, durante os meses de agosto e novembro no ano de 2024. A coleta de dados foi realizada pela pesquisadora principal deste estudo.

A intervenção educativa para os graduandos e mães adolescentes constou de compartilhamento de experiências prévias, dúvidas, comunicação clara e adaptação da linguagem para a realidade local, utilizando metodologias de ensino-aprendizagem ativas (oficina prática, simulação realística, *brainstorm* e exposição dialogada com vídeos), fundamentadas nos princípios da andragogia sob a luz da teoria da aprendizagem significativa de Auzubel^(12)^.

A 1^ª^ etapa, com duração de 02 horas, foi a Roda de conversa com os graduandos, quando disponibilizado o Guia Prático Para a Alta Hospitalar Segura de Recém-Nascidos elaborado previamente pelas pesquisadoras. Os graduandos foram incentivados a compartilhar ideias, para auxiliar na construção de conhecimentos úteis para as mães adolescentes que lhe garantissem a alta segura de seus filhos. A cada etapa se realizou uma avaliação e um *feedback* sobre o Guia Prático, com base em seus conhecimentos e experiências. Na semana seguinte deu-se a 2^ª^ Etapa, com duração de 03 horas, a Oficina de Simulação Realística com os graduandos, utilizando simulação com bonecos para demonstrar as informações que constavam no Guia Prático. Tentando reproduzir um ambiente acolhedor e funcional, semelhante ao lar das mães adolescentes, esta etapa proporcionou mudanças no guia e registro das narrativas. A 3^a^ Etapa, ocorreu em 02 dias com intervalo de 15 dias entre elas e duração de 02:10min e 02:40min respectivamente. Constou de Oficina de Simulação Realística agora com as mães adolescentes e foi conduzida pelos graduandos, sob supervisão da pesquisadora principal. O guia foi uma ferramenta dentro do processo de ensino-aprendizagem ativo, com prática simulada e seguida de reprodução dos cuidados aprendidos pelas adolescentes, com bonecos. Buscou-se promover o desenvolvimento de habilidades e construção do conhecimento das mães adolescentes. Esta etapa gerou a versão final do guia VAMOS PRA CASA, MAMÃE?, validado durante a fase de aprovação da dissertação de Mestrado da autora principal.

### Análise e Tratamento dos Dados

Para registro das narrativas dos participantes foi usado um gravador e, depois, transcritas as falas. Estas foram avaliadas por meio da análise de conteúdo, do tipo temática, seguindo as etapas: pré-análise, exploração do material, tratamento dos resultados obtidos e interpretação^(13)^. Após transcrição das narrativas gravadas e leituras exaustivas pelas pesquisadoras, foi elaborado o quadro – síntese para a tipificação e categorização temática.

### Aspectos Éticos

O estudo respeitou todos os preceitos éticos após ser aprovado pelo CEP, CAAE: - 71297523.10000.5013 e parecer provado nº 6.388.587, em 4 de outubro de 2023. Para trazer anonimato aos participantes, esses foram identificados por letras “E” para estagiárias e “A” para mães adolescentes seguidos de numeral cardinal (ex. E1, E2, A1, A2), quando referentes às discussões dos grupos.

## RESULTADOS E DISCUSSÃO

A análise incluiu 3 graduandas de enfermagem e 4 mães adolescentes (14–18 anos) durante a permanência de seus recém-nascidos na unidade hospitalar.

As análises das narrativas inferiram quatro categorias temáticas, a saber: 1 – Prática Reflexiva e Processo de ensino – aprendizagem; 2 – Estratégias para o processo educativo de mães adolescentes: abordagens lúdicas e humanizadas; 3 – Aprimoramento da prática de Enfermagem; 4 – Valorização Materna: *Feedback* como Ferramenta de Aprendizagem em Enfermagem.

### Categoria 1: Prática Reflexiva e Processo de Ensino – Aprendizagem

A aquisição de habilidades e competências na Enfermagem exigem que a aprendizagem no local de trabalho seja pautada na reflexão e compreensão do contexto das mães adolescentes assistidas. Para os graduandos, a interação direta com essas mães evidenciou a necessidade de adaptação dos conteúdos às especificidades individuais, promovendo um ensino sensível e ajustável.

Durante a intervenção educativa, desafios como a baixa escolaridade e condições socioeconômicas demandaram estratégias alternativas, como recursos visuais e simulações clínicas. Esses aspectos reforçam a importância da prática reflexiva, que inclui a compreensão dos determinantes sociais da saúde^(3)^.

A formação baseada na reflexão crítica e na experiência prática é essencial para desenvolver empatia e habilidades assistenciais, reduzindo o estresse materno e fortalecendo a autonomia dos pais^(14)^. A intervenção educativa implementada proporcionou uma compreensão mais profunda dos desafios enfrentados pelas mães adolescentes, permitindo que os graduandos aprimorassem sua capacidade de escuta ativa e adaptação ao contexto de cada família.

*Eu acho que é a habilidade de se adaptar à mãe e ao contexto que ela vive, o que ela tem em casa, inclusive… … nem sempre a realidade do que ensinamos é o que ela tem disponível em casa. (E3)*

As simulações clínicas foram fundamentais para o desenvolvimento da empatia e do compromisso social dos graduandos de Enfermagem, alinhando-se às diretrizes pedagógicas que promovem aprendizagem colaborativa e inclusiva^(15)^. A empatia, definida como a capacidade psicológica de compreender as emoções e realidades do outro, é uma competência essencial que deve ser desenvolvida por meio de treinamentos reflexivos^(16)^.

*Essa formação ajudou a pensar de que forma eu vou conversar com as pessoas a depender do seu nível social e de compressão cognitiva também. (E1). A gente precisa buscar outra forma de entender, até por conta do público que tem diversidade de compreensão. (E1)*

No contexto educacional, estudos apontam que a integração de atividades práticas nos currículos é eficaz para mitigar preconceitos e melhorar a interação com populações vulneráveis^(17)^. Destaca-se também que a aprendizagem no cenário real favorece o cuidado centrado na pessoa, aspecto essencial para a formação de enfermeiros^(18)^.

*A linguagem e o não julgamento são bem importantes, pois ela acha que não é capaz porque teve um filho nova[…] estarmos de prontidão […] (E2.) […]Se eu não tivesse feito a oficina, eu não ia saber aplicar e ensinar outras mães (E1). Permitir que ela seja um sujeito ativo, participando desse cuidado[…] (E3).*

O presente estudo reforçou que o ensino-aprendizagem na Enfermagem deve ser centrado no sujeito (no caso graduando e mãe adolescente), promovendo colaboração e construção de competências voltadas à humanização do cuidado. A motivação dos discentes é um fator essencial para a efetividade desse processo.

### Categoria 2: Estratégias para o Processo Educativo de Mães Adolescentes: Abordagens Lúdicas e Humanizadas

O desenvolvimento de habilidades empáticas e a adoção de estratégias educativas, adaptadas ao contexto de vida das mães adolescentes, são essenciais para uma prática reflexiva e um processo de ensino-aprendizagem eficaz. Isso ficou evidenciados neste estudo.

*[…] adolescente, ele está em constante mudança e a depender do estímulo que é feito que vai basear como ele vai agir ou não (E2).*

A experiência dos graduandos de Enfermagem revelou que um ambiente acolhedor e livre de julgamentos incentiva as mães a compartilharem suas dúvidas e dificuldades.

*Ficam pensando que vão ser julgadas se não fizerem certo. (E3).*

A pluralidade das experiências das participantes reforça a necessidade de adaptação das estratégias educativas, como exemplificado na criação de materiais visuais para facilitar a compreensão de mães com baixa escolaridade. Estudos indicam que a utilização de metodologias lúdicas e interativas, como simulações práticas com bonecos e outros materiais didáticos, favorece a aprendizagem significativa e a confiança das mães adolescentes nos cuidados com seus filhos^(19)^.

Ademais, estudos também reforçam a importância de desenvolver materiais educativos específicos para essa população, promovendo autonomia e segurança na tomada de decisão^(20)^.

*[…] A gente poderia fazer o desenho de um termômetro… Botar lá embaixo que o que estiver de verde está ok, o que estiver de vermelho ou diferente, é alerta. Porque aí ela já iria olhar para o termômetro e por mais que você não saiba ler, você vai comparar, ver se está igual (E2).*

A transição para a maternidade na adolescência é permeada por vulnerabilidades interseccionais que podem impactar a experiência materna, influenciada pelo espaço social ocupado pela mãe e pelo pai^(21)^. A rede de apoio, embora essencial, pode também dificultar a autonomia das mães adolescentes, como observado no relato de um dos graduandos de Enfermagem.

*Eu acho que a adolescente perde a função dela, que é de mãe, e a avó assume esse papel. A mãe dela e também mãe da criança, o que não poderia acontecer (E1).*

A implementação de Tecnologias Educacionais (TE) surge como uma alternativa eficaz para ampliar as possibilidades de educação em saúde, oferecendo materiais, estratégias que dinamizam o aprendizado e incentivam a participação ativa das adolescentes^(20)^. Metodologias tradicionais tendem a reforçar a passividade dos aprendizes, tornando-se necessário o uso de abordagens inovadoras que promovam interações mais dinâmicas e engajadoras^(19)^.

*Melhor é a prática para ela aprender fazendo. Para se sentir parte do processo. (E2). Elas são atentas aos detalhes (E3). E quanto mais se aplica mais se aprende. A cada nova oficina tem um aprendizado diferente. Uma mãe foi de um jeito e outra foi de outro jeito (E1).*

Os profissionais de enfermagem devem estar preparados para atender às necessidades específicas das adolescentes grávidas, oferecendo apoio, orientação e empatia^(22)^.

A pesquisa reforça a importância de práticas educativas humanizadas e interativas, que valorizem as experiências das mães adolescentes e fortaleçam seu protagonismo nos cuidados com seus filhos, promovendo maior confiança e autonomia durante a maternidade.

### Categoria 3: Aprimoramento da Prática

O aprimoramento da prática para os graduandos de Enfermagem envolvidos na intervenção educativa com mães adolescentes é um processo de construção mútua, no qual os futuros profissionais de enfermagem e as mães adolescentes desenvolveram suas aprendizagens em conjunto.

A participação dos graduandos em uma intervenção educativa sobre alta segura dos recém-nascidos, combinada com a aplicação de um guia prático, possibilitou a integração de conhecimentos prévios com novos conceitos, favorecendo uma aprendizagem significativa^(12)^.

O aprendizado teórico dos graduandos na intervenção foi complementado por simulações realísticas, fundamentais para a prática baseada em evidências, que reforçam as competências e habilidades dos graduandos, preparando-os para um atendimento.

*[…] A gente viu aqui o que é para ser feito, viu na cartilha como faz, viu como faz na prática, colocou em prática, assistiu o que a gente leu antes, já fez um link com aquilo ali e agora eu estou refazendo. Isso vai agregando mais conhecimento[…] (E3).*

A prática de Enfermagem fundamentada em evidências científicas integra as melhores ferramentas disponíveis, a experiência do educador e as necessidades dos pacientes^(23)^.

A simulação realística ajuda a preencher a lacuna entre o aprendizado teórico e a prática, o que melhora a compreensão. A interação com mães adolescentes foi um processo em que os graduandos adaptavam suas abordagens educacionais às necessidades específicas das mães, considerando seu contexto socioeconômico e emocional, o que reforçou suas competências de comunicação e empatia^(24)^.

*Então conhecer como o adolescente aprende, um pouco mais de neurociência. Faz com que a gente abra nossos olhos para entender o que acontece no cérebro deles, acolher e tentar chegar a eles de uma forma (E1).*

Assim, a formação em Enfermagem passa por um processo dinâmico de aprendizado, que visa à competência técnica e adaptações para diferentes cenários.

### Valorização Materna: Feedback Como Ferramenta de Aprendizagem em Enfermagem

A intervenção educativa com mães adolescentes realizada por meio de rodas de conversa e simulação realística favoreceu a valorização e a aprendizagem significativa sobre os cuidados com o recém-nascido. A abordagem de acolhimento, aliada ao uso de bonecos para simulação das práticas de alta segura, facilita a aquisição de habilidades pelas mães, promovendo confiança e autonomia desde o início da maternidade^(25)^.

*[…]porque, querendo ou não, a sensação de pertencimento vai dar empoderamento para que quando alguém em casa disser: “Não, menina! Não tem pra quê isso” …estejam capacitadas para mostrar que sabem (E3).*

Essa intervenção proporcionou um espaço de apoio e aprendizado que eliminou a insegurança e aumentou a autoconfiança das mães adolescentes^(26)^.

*Foi importante…E também pra mostrar para as pessoas de fora que eu sou capaz daquilo que eles pensam que eu não serei capaz (A3). Era bom também se fosse na alta (A2). Diz muita coisa que eu não sabia. Solteira ou casada, que não tinha muito apoio. Aí, vai lá e esqueça de alguma coisa, aí é bom, né? Ou até mãe de primeira viagem. É, mãe de primeira viagem, aí é que fica perdida (A3).*

O processo de educação em saúde fundamenta-se na conscientização do indivíduo que capacita as mães a agirem com segurança e autonomia^(27)^. Em revisão integrativa os autores refletiram sobre a importância da educação em saúde para mães adolescentes, destacando a necessidade de iniciar essa educação ainda no pré-natal, de forma individualizada, considerando o contexto social, cultural, emocional e educacional das mães, o que facilita a compreensão das informações e reduz o estresse parental^(28)^.

A utilização do *feedback* das mães adolescentes combinada com a supervisão docente foi essencial para ajustar o processo de ensino-aprendizagem, personalizando o cuidado e fortalecendo as competências nos estudantes de Enfermagem. Morin destaca que a educação em saúde deve ser reflexiva e transformadora, o que foi facilitada pela integração do conhecimento técnico com a realidade das mães adolescentes^(29)^.

*Era bom que fizesse sempre, porque é importante ficar relembrando, né? As coisas importantes (A3). Mas gostei de primeiro ler e depois fazer (A2).*

O *feedback* contínuo foi fundamental para aprimorar a prática docente e a compreensão dos cuidados^(30)^.

*Acho que é um conjunto. Nós estamos mais bem preparadas e com isso elas tem um nível de compreensão melhor (E2). Tanto profissional, educacional, quanto interpessoal. Você aprende a olhar o outro. Você vê o outro numa vulnerabilidade que você não imaginaria (E1).*

Os resultados do estudo mostraram que o diálogo contínuo entre mães adolescentes e graduandos foi fundamental para obter feedbacks e ajustar estratégias de ensino. O protagonismo do aprendiz foi enfatizado, contribuindo para um melhor desempenho dos graduandos e promovendo uma maior compreensão das mães adolescentes. Essas trocas foram essenciais para a adaptação das abordagens educacionais, resultando em um processo de ensino-aprendizagem mais eficaz e satisfatório para ambas as partes envolvidas.

### Limitações e Perspectivas

Este estudo apresenta limitações relacionadas ao fato de ter sido desenvolvido em um único serviço hospitalar, o que restringe a transferibilidade dos achados para outros contextos. Somam-se a isso possíveis vieses inerentes à pesquisa qualitativa, especialmente o viés de subjetividade, considerando que as intervenções foram conduzidas por graduandas de Enfermagem, cujas percepções e práticas são influenciadas por experiências prévias, expectativas e níveis de maturidade acadêmica. O envolvimento direto das graduandas no processo educativo e o vínculo profissional de parte das pesquisadoras com o setor podem ter contribuído para uma valorização positiva das experiências vivenciadas, caracterizando um possível viés de envolvimento.

A questão de gênero (só enfermeirandas presentes no estudo) é decorrente do fato da prevalência de mulheres no curso de Enfermagem. Para minimizar os vieses, adotaram-se estratégias como a permanência de apenas uma pesquisadora do setor durante as oficinas, a escuta prévia das graduandas antes da realização das intervenções, a utilização de espaço físico pedagógico fora da Unidade Neonatal e a supervisão dos recém-nascidos pela equipe de Enfermagem durante as atividades. A curta duração do estágio supervisionado também pode ter impactado a continuidade das intervenções e o acompanhamento longitudinal da aprendizagem das mães adolescentes. Como perspectivas futuras, recomenda-se a ampliação do tempo de acompanhamento, o uso de estratégias de triangulação de dados e a expansão das ações educativas para outros níveis de atenção à saúde, especialmente a Atenção Primária.

Em suma, os resultados apresentados não esgotam a problemática investigada, configurando-se como uma síntese provisória do fenômeno. As evidências colhidas no campo, em diálogo com a literatura, suscitam novos questionamentos que transcendem o escopo deste estudo. Sob o paradigma da pesquisa qualitativa, compreende-se que o conhecimento científico é construído mediante aproximações sucessivas, estabelecendo bases para que investigações futuras aprofundem as discussões aqui delineadas.

## CONCLUSÃO

O engajamento das mães no processo educativo aprimorou sua segurança nos cuidados com o recém-nascido e reforçou a importância da educação em saúde como ferramenta para empoderamento das adolescentes e transformação social.

Os achados deste estudo destacam a relevância de integrar metodologias inovadoras e tecnologias educacionais no currículo da Enfermagem, garantindo uma formação mais alinhada às demandas sociais. A simulação realística e o uso de materiais visuais demonstraram ser ferramentas valiosas na facilitação da aprendizagem, possibilitando aos futuros profissionais um preparo mais sólido para o cuidado materno-infantil. Dessa forma, a experiência dos graduandos evidenciou o impacto positivo da educação interativa na qualificação dos profissionais e na melhoria da assistência prestada.

A necessidade de continuidade e ampliação de pesquisas sobre práticas educativas voltadas para populações vulneráveis foi reforçada, especialmente entre mães adolescentes. A adaptação das estratégias de ensino a diferentes contextos socioculturais contribui para a formação de enfermeiros e também para a promoção de um cuidado mais inclusivo e eficaz. Associação entre teoria, prática e reflexão crítica pode ser crucial para melhorias no ensino da Enfermagem, garantindo profissionais capacitados para atuar com sensibilidade, ética e compromisso com a saúde materno-infantil.

## Data Availability

Conforme preconizado pela Ciência Aberta, o link do repositório no qual os dados de pesquisa foram depositados: https://famed.ufal.br/pt-br/pos-graduacao/ensino-na-saude/documentos/taac/2025/tacc_para_sigaa_23-05.pdf/view.

# Translation: Educación innovadora para madres adolescentes en cuidados neonatales: prácticas en la formación de enfermería*

**DOI:** 10.1590/1980-220X-REEUSP-2025-0499es

**Authors:** Maria Cecília Bandeira Arnaud Moura, Ana Maria Cavalcante Melo, Pedro Henrique do Nascimento Silva, Mércia Lamenha Medeiros

**Affiliations:** 1 Universidade Federal de Alagoas, Faculdade de Medicina, Maceió, AL, Brasil.; 2 Universidade Federal de Pernambuco, Recife, PE, Brasil.

**Keywords:** Salud Materno-Infantil;Educación en Enfermería, Capacitación Profesional, Madres Adolescentes

## Abstract

**Objetivo:**

Desarrollar las habilidades de los estudiantes de enfermería de pregrado para la educación sanitaria dirigida a madres adolescentes.

**Método:**

Estudio cualitativo, investigación-acción, hospital universitario público federal, con estudiantes de enfermería de pregrado en su rotación neonatal y con madres adolescentes. Las intervenciones se establecieron mediante la creación de una guía práctica para el alta segura, talleres de sensibilización para los estudiantes que se graduaban y sesiones educativas con las madres, grupos de debate y simulaciones realistas en un espacio adecuado, con la retroalimentación de los estudiantes que se graduaban y las madres. Los datos se analizaron mediante agrupaciones informativas, análisis temático y categorización.

**Resultados:**

Las estudiantes reconocieron la importancia de las iniciativas educativas para las madres adolescentes y el beneficio que estas aportan a su desarrollo. Las metodologías activas pueden fortalecer la práctica reflexiva y la formación en los programas de enfermería de pregrado, promoviendo estrategias lúdicas para la autoeficacia en el cuidado de madres adolescentes. La intervención promovió el empoderamiento de las madres adolescentes escuchando sus necesidades y fomentando su autonomía.

**Conclusión:**

Una educación innovadora e interactiva puede promover un aprendizaje significativo, mejorar las cualificaciones profesionales y alinear la enseñanza con las demandas sociales, con especial atención a la educación para la salud dirigida a madres adolescentes.

## INTRODUCCIÓN

La adolescencia comprende la transición entre la infancia y la vida adulta, una fase de descubrimientos y búsqueda de autonomía. Dicha fase puede traer una mayor susceptibilidad a situaciones de riesgo, influenciada tanto por aspectos objetivos como subjetivos en el contexto en el que viven los adolescentes (pueden llegar a ser más susceptibles a situaciones de riesgo, influenciados tanto por aspectos objetivos como subjetivos en el contexto en el que viven). Esta vulnerabilidad los expone a condiciones que comprometen la salud y a comportamientos de riesgo, como embarazos no deseados, sexo sin protección, infecciones y otras afecciones^(1)^.

Por lo tanto, justificar la vulnerabilidad únicamente por aspectos biológicos es muy reduccionista si no se toman en cuenta aspectos psicológicos y socioambientales. La asociación de la vulnerabilidad, simplemente, con la idea de rebeldía juvenil, puede reforzar el estereotipo público de hostilidad hacia los adolescentes. El modelo que debe seguir la salud debe permitir la discusión sobre comportamientos preventivos y el desarrollo de habilidades por parte de los adolescentes^(2)^.

Desde esta perspectiva y considerando la complejidad del proceso de la adolescencia, los profesionales de la salud que actúan a través de acciones de gestión, asistencia y educación, tanto a nivel individual como colectivo, en diferentes entornos, enfrentan desafíos constantes para desarrollar y aplicar estrategias de educación en salud que tengan como objetivo facilitar el proceso educativo con adolescentes^(3)^.

En el contexto de la licenciatura en Enfermería, es esencial que los estudiantes desarrollen competencias para actuar en la educación en salud, especialmente con poblaciones en situación de vulnerabilidad, como las madres adolescentes.

El aprendizaje significativo se produce cuando los nuevos conocimientos se integran en la estructura cognitiva del estudiante, volviéndose relevantes y aplicables en la práctica. Cabe destacar la importancia del diálogo y la problematización en el proceso educativo, enfatizando que la enseñanza debe ser un acto emancipador, en el que el educador y el educando aprenden juntos en un proceso continuo^(4)^.

En este contexto, los profesionales de enfermería en neonatología suelen lidiar con la singularidad de su práctica, marcada por una colaboración peculiar del área. La experiencia concreta en el manejo asistencial hace que la participación de este profesional sea fundamental en la formación, el intercambio de conocimientos y el desarrollo de habilidades que permitan buenas decisiones maternas en el cuidado de los recién nacidos^(5)^.

Se sabe que las orientaciones recibidas durante el periodo prenatal por las mujeres brasileñas destacan por ser orientaciones sobre maternidad de referencia, pero existe una desigualdad en la oferta de orientaciones^(6)^.

En el ámbito de la atención hospitalaria, las estrategias de educación en salud se dirigen al cuidado del bebé, la orientación sobre la lactancia materna y la salud de la mujer en el puerperio. La aplicación de estas estrategias durante el período de hospitalización puede contribuir a reducir la ansiedad de las madres adolescentes, especialmente de los bebés prematuros. Además, promueve una mayor confianza para la continuidad de los cuidados en el hogar, aumenta la adherencia al seguimiento ambulatorio y reduce la frecuencia de nuevas hospitalizaciones y/o fallecimientos^(7)^.

De acuerdo con este enfoque teórico, la presente investigación tuvo como objetivo desarrollar habilidades en estudiantes de grado de Enfermería para la educación en salud con madres adolescentes, como forma de convertir a estas madres en protagonistas del cuidado de sus propios hijos.

## MÉTODO

### Diseño del Estudio

Investigación de enfoque cualitativo, guiada por el método de investigación-acción. El diseño de investigación-acción, al ser un tipo de investigación social con base empírica, fue concebido y realizado en estrecha asociación con una acción o con la resolución de un problema colectivo, y en el que los investigadores y los participantes están involucrados de manera cooperativa o participativa. El análisis del objeto de identificación permite la construcción del conocimiento, y cuenta con todos los requisitos e instrumentos para ser considerado un constructo científico^(8)^.

### Lugar, Población y Criterios de Selección

La investigación se llevó a cabo en una Unidad Neonatal de la maternidad del Hospital Universitario, de carácter público federal, y contó con la participación de estudiantes de enfermería en prácticas curriculares supervisadas y madres adolescentes, de entre 14 y 18 años, que acompañaban a sus recién nacidos en la misma unidad hospitalaria. La muestra estuvo compuesta por estudiantes de pregrado en Enfermería que realizaban las Prácticas Curriculares Supervisadas (ECS) en un entorno hospitalario. La elección de este grupo se justificó por la inmersión intensiva de estas estudiantes en la práctica asistencial, dado que las prácticas comprenden una carga horaria de 500 horas, completadas en un semestre académico, conforme lo recomendado por las Directrices Curriculares Nacionales (DCN) para el curso de Enfermería^(9)^. Específicamente, las participantes seleccionaron la Unidad Neonatal como campo de práctica.

La asignación a esta unidad está limitada a tres plazas por semestre, y el proceso de selección se rige por criterios institucionales que tienen en cuenta la afinidad de la estudiante con el área y, de manera subsidiaria, el rendimiento académico como criterio de desempate. Las madres adolescentes ya habían recibido el alta obstétrica y acompañaban a sus recién nacidos hospitalizados; el tipo de parto no influyó en la participación, y todas estaban disponibles durante el período de hospitalización de sus hijos, que varió según la condición clínica.

Teniendo en cuenta la naturaleza de la investigación-acción y el escenario del estudio, se eligió como escenario la Unidad de Cuidados Intermedios Neonatales Canguro (UCINCA), que actualmente cuenta con 5 camas. La selección de la muestra se realizó respetando el proceso de saturación en las narrativas; este procedimiento permite definir el tamaño final de la muestra a partir del momento en que las nuevas inclusiones dejan de aportar información relevante al fenómeno investigado, interrumpiéndose así la captación de nuevos participantes y la realización de nuevos talleres^(10)^. La muestra en una investigación cualitativa debe estar vinculada a la dimensión del objeto (o de la indagación) que, a su vez, se articula con la elección del grupo o de los grupos a ser entrevistados y acompañados mediante observación participante^(11)^.

### Recolección de Datos

El desarrollo de la investigación se llevó a cabo a lo largo de tres meses, subdivididos en tres etapas, durante los meses de agosto y noviembre del año 2024. La recolección de datos fue realizada por la investigadora principal de este estudio.

La intervención educativa para los estudiantes de pregrado y las madres adolescentes consistió en el intercambio de experiencias previas, la resolución de dudas, la comunicación clara y la adaptación del lenguaje a la realidad local, utilizando metodologías activas de enseñanza-aprendizaje (taller práctico, simulación realista, *brainstorm* y exposición dialogada con videos), fundamentadas en los principios de la andragogía a la luz de la teoría del aprendizaje significativo de Auzubel^(12)^.

La primera etapa, con una duración de dos horas, consistió en una ronda de conversación con los estudiantes de pregrado, durante la cual se les proporcionó la Guía práctica para el alta hospitalaria segura de recién nacidos, elaborada previamente por las investigadoras. Se animó a los estudiantes de pregrado a compartir ideas, con el fin de contribuir a la construcción de conocimientos útiles para las madres adolescentes que les garantizaran el alta segura de sus hijos. En cada etapa se llevó a cabo una evaluación y se recabó *feedback* sobre la Guía práctica, basándose en sus conocimientos y experiencias. La semana siguiente tuvo lugar la segunda etapa, de tres horas de duración, el taller de simulación realista con los estudiantes de pregrado, utilizando simulaciones con muñecos para demostrar la información que figuraba en la Guía práctica. Con el objetivo de reproducir un ambiente acogedor y funcional, similar al hogar de las madres adolescentes, esta etapa propició cambios en la guía y el registro de las narrativas. La tercera etapa se llevó a cabo en dos días, con un intervalo de 15 días entre ellos y una duración de 2 horas y 10 minutos y 2 horas y 40 minutos, respectivamente. Consistió en un taller de simulación realista, ahora con las madres adolescentes, y fue dirigido por los estudiantes de pregrado, bajo la supervisión de la investigadora principal. La guía fue una herramienta dentro del proceso de enseñanza-aprendizaje activo, con práctica simulada seguida de la reproducción de los cuidados aprendidos por las adolescentes, con muñecos. Se buscó promover el desarrollo de habilidades y la construcción de conocimiento de las madres adolescentes. Esta etapa generó la versión final de la guía ¿VAMOS A CASA, MAMÁ?, validada durante la fase de aprobación de la tesis de maestría de la autora principal.

### Análisis y Tratamiento de los Datos

Para registrar las narrativas de los participantes se utilizó una grabadora y, posteriormente, se transcribieron los discursos. Estas fueron evaluadas mediante un análisis de contenido de tipo temático, siguiendo las etapas: preanálisis, exploración del material, tratamiento de los resultados obtenidos e interpretación^(13)^. Tras la transcripción de las narrativas grabadas y lecturas exhaustivas por parte de las investigadoras, se elaboró el cuadro de síntesis para la tipificación y categorización temática.

### Aspectos Éticos

El estudio respetó todos los preceptos éticos tras ser aprobado por el CEP, CAAE: - 71297523.10000.5013 y dictamen aprobado n.^º^ 6.388.587, el 4 de octubre de 2023. Para garantizar el anonimato de las participantes, estas fueron identificadas con la letra «E» para las estudiantes en prácticas y «A» para las madres adolescentes, seguidas de un número cardinal (p. ej., E1, E2, A1, A2), cuando se referían a las discusiones de los grupos.

## RESULTADOS Y DISCUSIÓN

El análisis incluyó a 3 estudiantes de enfermería y 4 madres adolescentes (14–18 años) durante la estancia de sus recién nacidos en la unidad hospitalaria.

Los análisis de las narrativas permitieron inferir cuatro cate­gorías temáticas, a saber: 1 – Práctica reflexiva y proceso de enseñanza-aprendizaje; 2 – Estrategias para el proceso educativo de las madres adolescentes: enfoques lúdicos y humanizados; 3 – Mejoramiento de la práctica de enfermería; 4 – Valorización materna: *Feedback* como herramienta de aprendizaje en enfermería.

### Categoría 1: Práctica Reflexiva y Proceso de Enseñanza-Aprendizaje

La adquisición de habilidades y competencias en enfermería exige que el aprendizaje en el lugar de trabajo se base en la reflexión y la comprensión del contexto de las madres adolescentes atendidas. Para los estudiantes de pregrado, la interacción directa con estas madres puso de manifiesto la necesidad de adaptar los contenidos a las especificidades individuales, promoviendo una enseñanza sensible y ajustable.

Durante la intervención educativa, desafíos como el bajo nivel educativo y las condiciones socioeconómicas exigieron estrategias alternativas, como recursos visuales y simulaciones clínicas. Estos aspectos refuerzan la importancia de la práctica reflexiva, que incluye la comprensión de los determinantes sociales de la salud^(3)^.

La formación basada en la reflexión crítica y la experiencia práctica es esencial para desarrollar empatía y habilidades asistenciales, reduciendo el estrés materno y fortaleciendo la autonomía de los padres^(14)^. La intervención educativa implementada proporcionó una comprensión más profunda de los desafíos que enfrentan las madres adolescentes, permitiendo que los estudiantes de pregrado mejoraran su capacidad de escucha activa y adaptación al contexto de cada familia.

*Creo que es la capacidad de adaptarse a la madre y al contexto en el que vive, lo que tiene en casa, incluso… … no siempre la realidad de lo que enseñamos es lo que ella tiene disponible en casa. (E3)*

Las simulaciones clínicas fueron fundamentales para el desarrollo de la empatía y el compromiso social de los estudiantes de Enfermería, alineándose con las directrices pedagógicas que promueven el aprendizaje colaborativo e inclusivo^(15)^. La empatía, definida como la capacidad psicológica de comprender las emociones y realidades del otro, es una competencia esencial que debe desarrollarse mediante entrenamientos reflexivos^(16)^.

*Esta formación me ayudó a pensar en cómo voy a hablar con las personas dependiendo de su nivel social y también de su comprensión cognitiva. (E1). Necesitamos buscar otra forma de entender, incluso debido a que el público tiene una diversidad de comprensión. (E1)*

En el contexto educativo, los estudios señalan que la integración de actividades prácticas en los planes de estudio es eficaz para mitigar los prejuicios y mejorar la interacción con poblaciones vulnerables^(17)^. También se destaca que el aprendizaje en el escenario real favorece la atención centrada en la persona, un aspecto esencial para la formación de enfermeros^(18)^.

*El lenguaje y la ausencia de juicios son muy importantes, ya que ella cree que no es capaz porque tuvo un hijo joven […] estar preparados […] (E2.) […] Si no hubiera hecho el taller, no sabría cómo aplicar y enseñar a otras madres (E1). Permitirle ser un sujeto activo, participando en ese cuidado […] (E3).*

El presente estudio reforzó que la enseñanza-aprendizaje en Enfermería debe centrarse en el sujeto (en este caso, la estudiante y la madre adolescente), promoviendo la colaboración y la construcción de competencias orientadas a la humanización del cuidado. La motivación de los estudiantes es un factor esencial para la eficacia de este proceso.

### Categoría 2: Estrategias para el Proceso Educativo de las Madres Adolescentes: Enfoques Lúdicos y Humanizados

El desarrollo de habilidades empáticas y la adopción de estrategias educativas, adaptadas al contexto de vida de las madres adolescentes, son esenciales para una práctica reflexiva y un proceso de enseñanza-aprendizaje eficaz. Esto quedó evidenciado en este estudio.

*[…] adolescente, está en constante cambio y depende del estímulo que se le brinde para determinar cómo actuará o no (E2).*

La experiencia de los estudiantes de enfermería reveló que un ambiente acogedor y libre de juicios anima a las madres a compartir sus dudas y dificultades.

*Piensan que serán juzgadas si no lo hacen bien. (E3).*

La diversidad de experiencias de las participantes refuerza la necesidad de adaptar las estrategias educativas, como se ejemplifica en la creación de materiales visuales para facilitar la comprensión de las madres con bajo nivel educativo. Los estudios indican que el uso de metodologías lúdicas e interactivas, como simulaciones prácticas con muñecos y otros materiales didácticos, favorece el aprendizaje significativo y la confianza de las madres adolescentes en el cuidado de sus hijos^(19)^.

Además, los estudios también refuerzan la importancia de desarrollar materiales educativos específicos para esta población, promoviendo la autonomía y la seguridad en la toma de decisiones^(20)^.

*[…] Podríamos hacer el dibujo de un termómetro… Poner ahí abajo que lo que esté en verde está bien, lo que esté en rojo o diferente, es una alerta. Porque así ella ya miraría el termómetro y, aunque no sepa leer, compararía, vería si está igual (E2).*

La transición a la maternidad en la adolescencia está marcada por vulnerabilidades interseccionales que pueden afectar la experiencia materna, influenciada por el espacio social que ocupan la madre y el padre^(21)^. La red de apoyo, aunque esencial, también puede dificultar la autonomía de las madres adolescentes, como se observa en el relato de uno de los estudiantes de Enfermería.

*Creo que la adolescente pierde su función, que es la de madre, y la abuela asume ese papel. Su madre y también la madre del niño, lo que no debería suceder (E1).*

La implementación de Tecnologías Educativas (TE) surge como una alternativa eficaz para ampliar las posibilidades de la educación en salud, ofreciendo materiales y estrategias que dinamizan el aprendizaje e incentivan la participación activa de las adolescentes^(20)^. Las metodologías tradicionales tienden a reforzar la pasividad de los alumnos, por lo que se hace necesario el uso de enfoques innovadores que promuevan interacciones más dinámicas y atractivas^(19)^.

*Es mejor la práctica para que ella aprenda haciendo. Para sentirse parte del proceso. (E2). Ellas están atentas a los detalles (E3). Y cuanto más se aplica, más se aprende. En cada nuevo taller hay un aprendizaje diferente. Una madre fue de una manera y otra fue de otra manera (E1).*

Los profesionales de enfermería deben estar preparados para atender las necesidades específicas de las adolescentes embarazadas, ofreciendo apoyo, orientación y empatía^(22)^.

La investigación refuerza la importancia de prácticas educativas humanizadas e interactivas, que valoren las experiencias de las madres adolescentes y fortalezcan su protagonismo en el cuidado de sus hijos, promoviendo mayor confianza y autonomía durante la maternidad.

### Categoría 3: Perfeccionamiento de la Práctica

El perfeccionamiento de la práctica para los estudiantes de enfermería involucrados en la intervención educativa con madres adolescentes es un proceso de construcción mutua, en el que los futuros profesionales de la enfermería y las madres adolescentes desarrollaron su aprendizaje de manera conjunta.

La participación de los estudiantes en una intervención educativa sobre el alta segura de los recién nacidos, combinada con la aplicación de una guía práctica, permitió la integración de conocimientos previos con nuevos conceptos, lo que favoreció un aprendizaje significativo^(12)^.

El aprendizaje teórico de los estudiantes de pregrado en la intervención se complementó con simulaciones realistas, fundamentales para la práctica basada en la evidencia, que refuerzan las competencias y habilidades de los estudiantes, preparándolos para la atención.

*[…] Aquí vimos lo que hay que hacer, vimos en el manual cómo se hace, vimos cómo se hace en la práctica, lo pusimos en práctica, observamos lo que habíamos leído antes, ya establecimos un vínculo con eso y ahora lo estoy repitiendo. Esto va sumando más conocimiento[…] (E3)*.

La práctica de la enfermería basada en la evidencia científica integra las mejores herramientas disponibles, la experiencia del educador y las necesidades de los pacientes^(23)^.

La simulación realista ayuda a cerrar la brecha entre el aprendizaje teórico y la práctica, lo que mejora la comprensión. La interacción con madres adolescentes fue un proceso en el que los estudiantes de pregrado adaptaban sus enfoques educativos a las necesidades específicas de las madres, considerando su contexto socioeconómico y emocional, lo que reforzó sus competencias de comunicación y empatía^(24)^.

*Entonces, conocer cómo aprende el adolescente, un poco más de neurociencia. Nos hace abrir los ojos para entender lo que pasa en sus cerebros, acogerlos e intentar llegar a ellos de una manera (E1).*

Así, la formación en Enfermería pasa por un proceso dinámico de aprendizaje, que apunta a la competencia técnica y a las adaptaciones para diferentes escenarios.

### Valoración Materna: la Retroalimentación como Herramienta de Aprendizaje en Enfermería

La intervención educativa con madres adolescentes, llevada a cabo mediante círculos de conversación y simulaciones realistas, favoreció la valorización y el aprendizaje significativo sobre el cuidado del recién nacido. El enfoque de acogida, junto con el uso de muñecos para simular prácticas de alta segura, facilita la adquisición de habilidades por parte de las madres, promoviendo la confianza y la autonomía desde el inicio de la maternidad^(25)^.

*[…]porque, lo queramos o no, el sentido de pertenencia les dará empoderamiento para que cuando alguien en casa diga: «¡No, niña! No hay por qué hacer eso»… estén capacitadas para demostrar que saben (E3).*

Esta intervención proporcionó un espacio de apoyo y aprendizaje que eliminó la inseguridad y aumentó la autoconfianza de las madres adolescentes^(26)^.

*Fue importante… Y también para demostrarle a la gente de afuera que soy capaz de lo que ellos piensan que no seré capaz (A3). También sería bueno si fuera al dar de alta (A2). Dice muchas cosas que yo no sabía. Soltera o casada, que no tenía mucho apoyo. Entonces, vas y te olvidas de algo, ahí es bueno, ¿no? O incluso madre primeriza. Sí, madre primeriza, ahí es donde se siente perdida (A3).*

El proceso de educación en salud se basa en la concienciación del individuo, lo que capacita a las madres para actuar con seguridad y autonomía^(27)^. En una revisión integrativa, los autores reflexionaron sobre la importancia de la educación en salud para las madres adolescentes, destacando la necesidad de iniciar esta educación ya en el período prenatal, de manera individualizada, considerando el contexto social, cultural, emocional y educativo de las madres, lo que facilita la comprensión de la información y reduce el estrés parental^(28)^.

El uso de la retroalimentación de las madres adolescentes, combinado con la supervisión docente, fue esencial para ajustar el proceso de enseñanza-aprendizaje, personalizando la atención y fortaleciendo las competencias de los estudiantes de enfermería. Morin destaca que la educación en salud debe ser reflexiva y transformadora, lo cual se vio facilitado por la integración del conocimiento técnico con la realidad de las madres adolescentes^(29)^.

*Sería bueno que lo hiciera siempre, porque es importante seguir recordando, ¿no? Las cosas importantes (A3). Pero me gustó leer primero y luego hacerlo (A2).*

La retroalimentación continua fue fundamental para mejorar la práctica docente y la comprensión de los cuidados^(30)^.

*Creo que es un conjunto. Estamos mejor preparadas y, con eso, ellas tienen un mejor nivel de comprensión (E2). Tanto a nivel profesional, educativo como interpersonal. Uno aprende a mirar al otro. Uno ve al otro en una vulnerabilidad que no se imaginaría (E1).*

Los resultados del estudio mostraron que el diálogo continuo entre las madres adolescentes y los estudiantes universitarios fue fundamental para obtener retroalimentación y ajustar las estrategias de enseñanza. Se enfatizó el protagonismo del alumno, lo que contribuyó a un mejor desempeño de los estudiantes universitarios y promovió una mayor comprensión de las madres adolescentes. Estos intercambios fueron esenciales para la adaptación de los enfoques educativos, lo que dio lugar a un proceso de enseñanza-aprendizaje más eficaz y satisfactorio para ambas partes involucradas.

### Limitaciones y Perspectivas

Este estudio presenta limitaciones relacionadas con el hecho de haberse desarrollado en un único servicio hospitalario, lo que restringe la transferibilidad de los hallazgos a otros contextos. A esto se suman posibles sesgos inherentes a la investigación cualitativa, especialmente el sesgo de subjetividad, considerando que las intervenciones fueron realizadas por estudiantes de enfermería, cuyas percepciones y prácticas están influenciadas por experiencias previas, expectativas y niveles de madurez académica. La participación directa de las estudiantes en el proceso educativo y el vínculo profesional de parte de las investigadoras con el sector pueden haber contribuido a una valoración positiva de las experiencias vividas, lo que caracteriza un posible sesgo de implicación.

La cuestión de género (solo estudiantes de enfermería presentes en el estudio) se debe a la prevalencia de mujeres en la carrera de Enfermería. Para minimizar los sesgos, se adoptaron estrategias como la presencia de una sola investigadora del sector durante los talleres, la escucha previa de las estudiantes de pregrado antes de realizar las intervenciones, el uso de un espacio físico pedagógico fuera de la Unidad Neonatal y la supervisión de los recién nacidos por parte del equipo de enfermería durante las actividades. La corta duración de la pasantía supervisada también puede haber afectado la continuidad de las intervenciones y el seguimiento longitudinal del aprendizaje de las madres adolescentes. Como perspectivas futuras, se recomienda ampliar el tiempo de seguimiento, utilizar estrategias de triangulación de datos y extender las acciones educativas a otros niveles de atención de la salud, especialmente a la Atención Primaria.

En resumen, los resultados presentados no agotan la problemática investigada, configurándose como una síntesis provisional del fenómeno. Las evidencias recogidas en el campo, en diálogo con la literatura, suscitan nuevos cuestionamientos que trascienden el alcance de este estudio. Bajo el paradigma de la investigación cualitativa, se entiende que el conocimiento científico se construye mediante aproximaciones sucesivas, estableciendo bases para que futuras investigaciones profundicen en las discusiones aquí delineadas.

## CONCLUSIÓN

La participación de las madres en el proceso educativo mejoró su seguridad en el cuidado del recién nacido y reforzó la importancia de la educación en salud como herramienta para el empoderamiento de las adolescentes y la transformación social.

Los hallazgos de este estudio destacan la relevancia de integrar metodologías innovadoras y tecnologías educativas en el plan de estudios de Enfermería, garantizando una formación más alineada con las demandas sociales. La simulación realista y el uso de materiales visuales demostraron ser herramientas valiosas para facilitar el aprendizaje, lo que permitió a los futuros profesionales una preparación más sólida para la atención materno-infantil. De esta manera, la experiencia de los estudiantes de pregrado evidenció el impacto positivo de la educación interactiva en la cualificación de los profesionales y en la mejora de la asistencia prestada.

Se reforzó la necesidad de continuar y ampliar las investigaciones sobre prácticas educativas dirigidas a poblaciones vulnerables, especialmente entre las madres adolescentes. La adaptación de las estrategias de enseñanza a diferentes contextos socioculturales contribuye a la formación de enfermeros y también a la promoción de una atención más inclusiva y eficaz. La asociación entre teoría, práctica y reflexión crítica puede ser crucial para mejorar la enseñanza de la enfermería, garantizando profesionales capacitados para actuar con sensibilidad, ética y compromiso con la salud materno-infantil.

## Data Availability

De acuerdo con lo recomendado por la Ciencia Abierta, el enlace al repositorio en el que se depositaron los datos de la investigación: https://famed.ufal.br/pt-br/pos-graduacao/ensino-na- saude/documentos/taac/2025/tacc_para_sigaa_23-05.pdf/view.
